# Polydopamine Nanosystems in Drug Delivery: Effect of Size, Morphology, and Surface Charge

**DOI:** 10.3390/nano14030303

**Published:** 2024-02-01

**Authors:** Arianna Menichetti, Dario Mordini, Marco Montalti

**Affiliations:** Department of Chemistry “Giacomo Ciamician”, University of Bologna, Via Selmi 2, 40126 Bologna, Italy; arianna.menichetti2@unibo.it (A.M.); dario.mordini2@unibo.it (D.M.)

**Keywords:** drug delivery, nanocarrier, polydopamine, targeted delivery, disease treatment, cancer therapy

## Abstract

Recently, drug delivery strategies based on nanomaterials have attracted a lot of interest in different kinds of therapies because of their superior properties. Polydopamine (PDA), one of the most interesting materials in nanomedicine because of its versatility and biocompatibility, has been widely investigated in the drug delivery field. It can be easily functionalized to favor processes like cellular uptake and blood circulation, and it can also induce drug release through two kinds of stimuli: NIR light irradiation and pH. In this review, we describe PDA nanomaterials’ performance on drug delivery, based on their size, morphology, and surface charge. Indeed, these characteristics strongly influence the main mechanisms involved in a drug delivery system: blood circulation, cellular uptake, drug loading, and drug release. The understanding of the connections between PDA nanosystems’ properties and these phenomena is pivotal to obtain a controlled design of new nanocarriers based on the specific drug delivery applications.

## 1. Introduction

**Figure 1:**
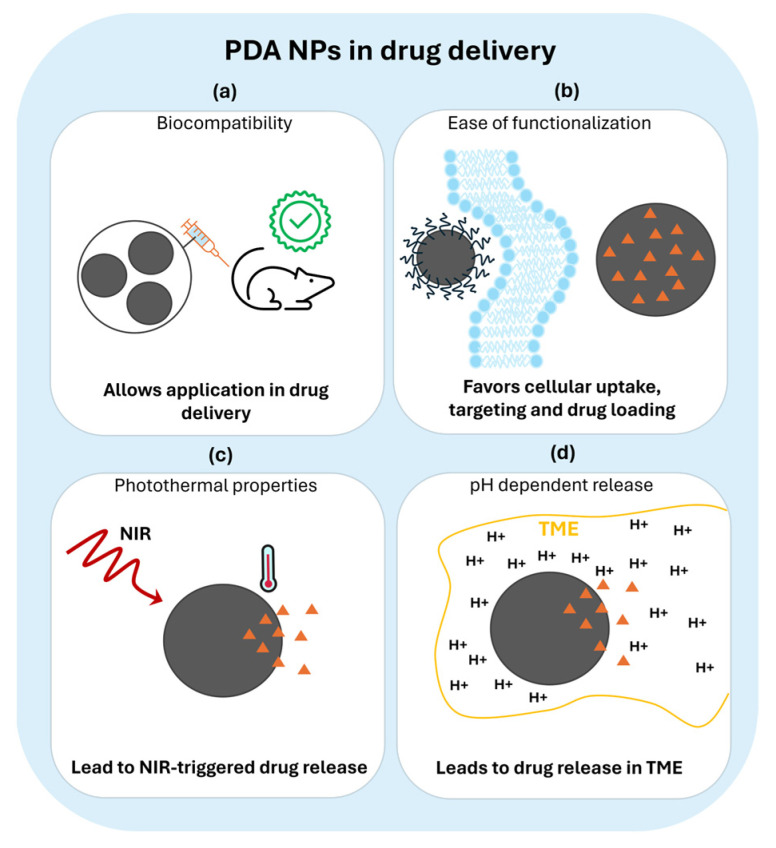
How PDA properties influence drug delivery requisites. The drug to be released is represented by an orange triangle. (**a**) Biocompatibility is the main requisite for application in drug delivery, (**b**) the ease of functionalization favors cellular uptake and drug loading, (**c**) photothermal behavior, obtained by NIR (near-infrared) light irradiation, triggers drug release, (**d**) pH-dependent drug release allows for controlled release only at under acidic pH conditions of the tumor microenvironment (TME).

Nanotechnology-based drug delivery strategies have recently attracted tremendous attention. Indeed, the use of traditionally sized agents has several drawbacks, such as low in vivo stability and bioavailability, poor body absorption and cell internalization, and issues with targeted delivery [1], while the use of nanotechnology leads to advantages in terms of pharmacokinetics and release efficiency [2]. First of all, nanocarriers have better stability and circulation in the human body [3,4] and allow for largely improved cellular uptake [5,6]. Moreover, drugs can effectively be loaded into the nanosystems in different ways, such as adsorption, encapsulation, or binding to nanoparticles’ (NPs) surface functionalities; this can lead to co-delivery of multiple drugs and to a stimuli-controlled drug release [7,8,9]. These properties overcome the issue of non-specificity, which is one of the biggest issues for drug delivery, especially in anticancer therapy that aims to eliminate toxic agents in normal cells [10,11]. Finally, another advantage of using nanocarrier-based therapy is the possibility of developing theranostic systems [12]. Indeed, it is often feasible to combine NPs’ drug encapsulation capabilities with their use as imaging agents by exploiting NPs’ intrinsic properties such as fluorescence [13,14,15] or by further modifying them with imaging-related agents, like in the case of lanthanide doping for magnetic resonance imaging (MRI) [16,17,18]. Currently, nanocarriers are in continuous development, and we can delineate four classes of NPs used for drug delivery purposes: lipid-based NPs, metal–organic frameworks (MOFs), inorganic NPs, and polymeric NPs [19,20]. Among lipidic NPs, liposomes are the most commonly used for drug release. In fact, they are biocompatible spherical vesicles made by non-toxic phospholipids and characterized by hydrophobic and hydrophilic areas [21]; thus, they are easily exploitable as drug carriers. In this field, liposomes have been widely studied for decades in the delivery of anti-cancer, anti-fungal, and antibiotic drugs and are the first systems to have made the transition from concept to clinical applications [22]. Indeed, liposomal doxorubicin (LD-Doxil and Myocet) was the first drug delivery system approved for cancer treatment [10,23,24]. MOFs, thanks to their high surface area and their adjustable pore size, are ideal platforms for drug encapsulation and controlled release [20,25]. Moreover, their biodegradability is an added value for the application of these materials as nanocarriers in nanomedicine [26]. Inorganic NPs, mainly based on silica, gold, and iron, have also been largely used for drug delivery [19]. Indeed, inorganic NPs are quite versatile because they can be designed with varying sizes and morphologies, as in the case of gold nanosystems, which can be synthesized as nanospheres, nanorods, nanoshells, and nanocages [27,28,29,30]. Moreover, inorganic nanocarriers can have additional properties that are dependent on the material they are composed of. For example, gold nanorods are often characterized by photothermal properties and iron NPs by magnetic properties [31,32]. The third class of NPs used in drug delivery systems is polymeric NPs. They are advantageous because they are obtained using synthetic or natural precursors through several methods, with high biocompatibility [33,34,35]. Other important features of polymeric NPs are the various possibilities of drug encapsulation, including embedding in the NP’s core, entrapment in or conjugation to the polymer matrix, and functionalization onto the surface. Among polymeric NPs, polydopamine (PDA) is one of the most investigated materials for applications in nanomedicine. PDA is the synthetic analogue of melanin, a natural polymer found in many living organisms that can have different roles based on its peculiar properties [36,37,38,39]. PDA is obtained by the oxidative self-polymerization of precursors, such as dopamine or L-dopa [40], and presents many interesting properties, which makes it a very promising material for use in biomedicine. One of the main reasons that suggests PDA and melanin-like materials for use in this field is their excellent and widely investigated biocompatibility [41]. Indeed, PDA is not only intrinsically biocompatible but also promotes cell adhesion to the surface functionalized with it [42,43]. Thanks to these properties, PDA is often used in combination with toxic nanomaterials, for example, gold NPs [44] or quantum dots [45], to develop harmless systems for cells [46]. Another important property of PDA is its ease of functionalization due to the presence of phenol, imine, and amine groups that allow for efficient molecular binding or ion anchoring [47]. Finally, PDA presents other interesting features for biomedical applications, such as efficient photothermal and antioxidant properties [48,49,50]. The combination of all these PDA characteristics makes it a very promising platform for drug delivery purposes and solves many issues related to this topic, as depicted in Figure 1.

**Figure 2:**
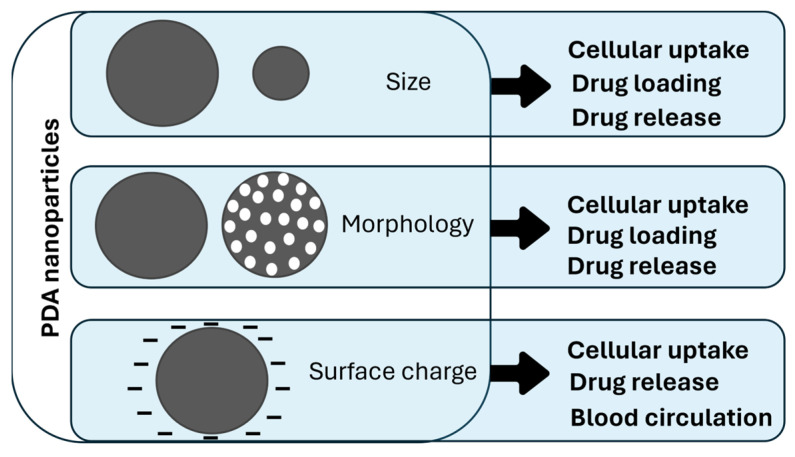
Influence of size, morphology, and surface charge of PDA NPs on drug delivery-related processes. The influence that each property has on cellular uptake, drug loading, drug release, and blood circulation is highlighted by arrows.

As shown in Figure 1, some PDA properties are particularly advantageous for drug delivery. First, its ease of functionalization allows for the improvement of the cellular uptake properties and opens the potential for an efficient drug encapsulation. Moreover, the possibility of PDA functionalization allows for the enhancement of its therapeutic efficiency by surface modification with drug active targeting moieties, such as nucleic acids, peptides, and antibodies [51,52]. Also, the peculiar properties of PDA can be exploited in combination with other systems, in PDA-coated organic and inorganic materials [52]. For example, PDA coating on Ag-Au NPs increases the efficiency of their photothermal activity [53]. Secondly, the photothermal properties of PDA can lead to systems with controlled drug release; many PDA-based nanocarriers have a strong drug release in response to NIR light irradiation [54]. Another factor that leads to a controlled release is the pH-dependent behavior of PDA NPs. The protonation of the amine groups, which are usually the binding point of the encapsulated drugs, triggers the delivery because of the weakening of the chemical interaction between the NPs and dopants [55]. This makes possible a release which is mainly confined in the tumor microenvironment, at almost pH 5.0, and is more limited during blood circulation (pH 7.4) [55]. Having two options of controlled release using the same material (NIR and pH-controlled release) leads to the development of dual-stimuli drug delivery. The above-described features make PDA a widely studied nanocarrier; indeed, PDA drug loading, release, and pharmacokinetic behavior can be influenced by each system’s specific properties. In this review, our aim is to identify and describe PDA NPs’ behavior based on size, morphology, and surface charge (Figure 2).

These parameters are always essential for understanding the behavior of PDA-based systems because they strongly influence, in different ways, drug loading and release, cellular uptake, and blood circulation. As depicted in Figure 2, size and morphology variations usually affect cellular uptake mechanisms and the amount of the loaded and released drug, since these mechanisms are dependent on the surface-to-volume ratio of the material. Surface charge has a role in cellular uptake and blood circulation and is also involved in drug release mechanisms. Investigating these aspects can provide a more focused design of PDA-based nanocarriers with the aim of strongly improving their performances as drug delivery systems.

## 2. Size Influence on Drug Delivery

**Figure 3:**
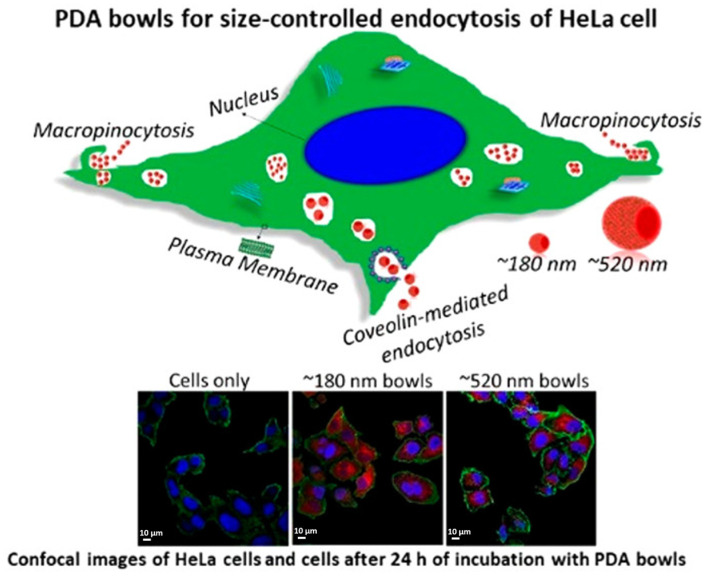
Uptake mechanism of 180 nm and 520 nm PDA bowls in Hela cells and confocal images of HeLa cell comparison of the two bowls, after 24 h of incubation. Reprinted with permission from ref [60]. Copyright 2021, American Chemical Society.

Size is one of the parameters that most influences drug delivery processes; indeed, it has a relevant role in cellular uptake, drug loading, and drug release. Cellular uptake is an important part of pharmacokinetics, strongly influenced by the size of the system but also connected to biodistribution [56], blood circulation [57], and tumor permeability [58] and dependent on surface charge and cell types [59]. Thus, distinguishing the size effect from other parameter effects in these complex mechanisms is quite challenging. This is probably why there are very few studies on the size-dependent behavior of PDA NPs on this topic. One recent interesting study is given by Acter and co-workers, in which they investigated the behavior of bowl-shaped mesoporous PDA NPs (MPDA NPs) of different sizes in the internalization mechanism of human cervical carcinoma epithelial cells (HeLa cells) [60]. They synthesized bowl-shaped MPDA NPs, tuning the size by modifying reactant concentration, polymerization time, and pH during the reaction. After having investigated the modification of these parameters, they selected 180 nm and 520 nm bowls, which had a significant difference in size, to study the uptake mechanism in HeLa cells, as shown in Figure 3.

**Figure 4:**
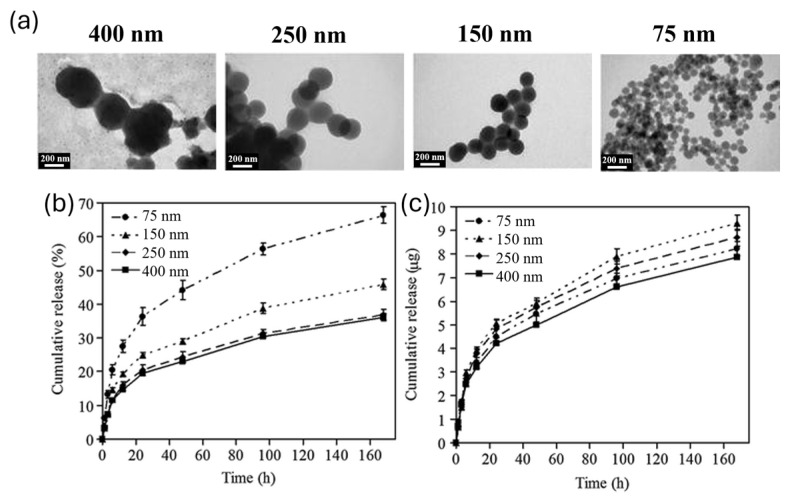
(**a**) TEM images of the PDA NPs with different sizes: (**b**) release percent and (**c**) release amount of CPT from the different PDA NPs at 37 °C and at pH 7.4. Adapted with permission from ref [65]. Copyright 2013, Springer Nature.

They incubated the bowls in HeLa cells for 4 h and 24 h, measuring the PDA quantity by flow cytometry. The results showed a faster internalization of smaller bowls (180 nm). This effect is often observed in nanosystem uptake, and it could be correlated with a higher adhesion to cell surface, given by higher aspect ratio [61]. Then, the uptake mechanism of differently sized NPs was investigated: the behavior of 180 nm and 520 nm bowls in HeLa Cells was tested in the presence of inhibitors for different internalization pathways. At first, cells were treated with sodium azide, which inhibited ATP synthesis [62]; internalization was significatively reduced for both sizes, denoting that the processes these nanosystems were undergoing were energy dependent. Afterwards, macropinocytosis and caveolae-mediated endocytosis pathways were investigated, using cytochalasin D (CytD) and genistein to, respectively, inhibit the former and the latter. In the cells treated with CytD, the internalization of 180 nm bowls was blocked, displaying their tendency to enter the cell by macropynocitosis, while no difference was observed for 520 nm bowls. Instead, genistein treatment led to the uptake inhibition of 520 nm bowls, revealing its dependence on caveolae-mediated endocytosis, and did not have an effect on 180 nm NPs. These results showed that smaller NPs are preferentially internalized through macropinocytosis and bigger ones through caveolae-mediated endocytosis. NP size affects not only cell uptake mechanisms, but also drug loading and drug release efficiency, essential aspects to design drug delivery nanosystems. In general, drug loading tends to increase with size, while drug release tends to be faster with a decrease in size [63]. Size-dependent behavior of drug release is due to the higher surface area-to-volume ratio of smaller NPs, which hastens their degradation. On the contrary, larger NPs manage to maintain the loaded drug for more time [63,64]. An example of this behavior in PDA NPs is reported in the paper of Ho et al.; PDA NPs of different sizes were synthesized, varying the initial pH of the reaction mixture and the reaction time [65]. They obtained NPs of 400 nm, 250 nm, 150 nm, and 75 nm and tested the loading and release of camptothecin (CPT) as a model of anticancer drug (Figure 4). For 1 mg of PDA NPs, 10.85, 11.81, 10.17, and 6.19 µg of CPT were loaded, respectively, for 400 nm, 250 nm, 150 nm, and 75 nm NPs. According to the authors, the reason the loading in 400 nm NPs was lower than the one in 250 nm NPs depended on the wider size distribution of 400 nm NPs. The CPT release, observed in vitro during the first day, was observed to be 19, 20, 25, and 36%, respectively, for 400 nm, 250 nm, 150 nm, and 75 nm NPs, showing also in this case the expected trend of faster release with smaller sizes, as shown in Figure 4.

Considering the size-dependent behavior of PDA NPs in drug delivery, it is important to highlight that it is convenient to have a release that is not too slow, but also, a release that is too fast is not favorable because it can cause drug leaking, leading to a lack of bioavailability [64]. According to this, we can notice that recent papers on PDA NPs with effective drug delivery performances usually show an NP size which is, on average, between 100 and 150 nm. For example, Li et al. developed PDA NPs, functionalized with targeting peptides for tumor sites (PDA-RGDC), for pH and NIR controlled release of Doxorubicin (DOX), coupled with photoacoustic (PA) imaging and photothermal therapy (PTT) performances [66]. They obtained, with an oxidation and self-polymerization method, 120 nm PDA NPs, whose size further increased after the addition of the peptide functionalities. DOX can effectively bind to PDA by means of hydrophobic, π-π stacking interactions or hydrogen bonding [67,68], and in this work, its loading in PDA NPs was studied by soaking PDA-RGDC and DOX in PBS (pH 7.4), choosing a 1:1 PDA-RGDC/DOX sample for drug delivery tests. When tested in HeLa Cells, loaded particles displayed a selective accumulation in the nucleus and stability up to 48 h, with only 5% of drug release at pH 7. This allowed for the control of drug delivery by pH and NIR irradiation stimuli that resulted in effective DOX release, demonstrated both in vitro and in vivo. Cao et al. also developed an anticancer nanosystem embedding gold nanorods (GNRs), functionalized with DOX for chemotherapy and chlorine6 (Ce6) for photodynamic therapy, in PDA NPs. PDA scaffolds were synthesized by dopamine self-polymerization leading to a size of almost 126 nm, which was increased to almost 170 nm after the functionalization with DOX-modified GNRs and Ce6. These NPs were expected to reach the tumor site by means of enhanced permeability and retention effect (EPR), which represents a typical accumulation driving force. Indeed, the EPR effect is caused by the tumor structure, which presents wide fenestrations in its vasculatures and allows nanocarriers to enter the tumor site more easily [69,70]. In particular, it has been demonstrated that NPs with sizes between 100 and 200 nm are the best for the EPR effect [58,71,72], in accordance with the size obtained in the paper. DOX loading in the system was reported to be 10%. At pH 7.4 and 5.5, DOX release in the first 2 h was only 6.54% and 7.86%, respectively. The situation drastically changed in the presence of NIR light irradiation: after three NIR irradiation cycles, the quantities of DOX released were 57.59% at pH 7.4 and 83.68% at pH 5.5. These results allowed the system to be controlled by NIR light, both at neutral and acidic pH. NP and DOX accumulation in HeLa cells was mainly observed in the cytoplasm for 4 h and increased with incubation time. Instead, under NIR light irradiation, the DOX fluorescence signal was not limited to the cytoplasm but was observed in all the cells, denoting good photorelease efficiency. As reported in these examples, the first step in the development of PDA nanocarriers is always the optimization of the synthesis conditions that lead to the optimal size, which is always between 100 nm and 200 nm. This size is probably big enough to obtain a significative drug loading and controlled release and small enough to be effectively internalized both in the tumor site (through EPR) and in the cells (through macrocytosis and endocytosis). However, this analysis is not sufficient to tailor defined PDA-related size effects; further investigation and direct comparison among differently sized PDA NPs in the drug delivery field are required.

## 3. Morphology Role in Drug Delivery

**Figure 5:**
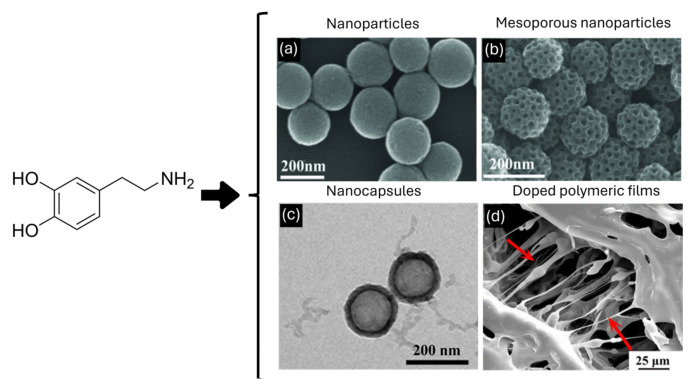
SEM images of (**a**) PDA NPs, (**b**) MPDA NPs; (**a**,**b**) adapted with permission from ref [83], copyright 2014, John Wiley and Sons. (**c**) TEM image of PDA nanocapsules [90] and (**d**) the structure of PDA-clay-PAM hydrogel, arrows indicating microfibril structure, adapted with permission from ref [89], copyright 2017, American Chemical Society.

Since the very beginning of nanoscience and nanotechnology, researchers had proof that not only does the size of matter influence its properties, moving from the macro- to the nano-world, but also the shape/morphology has a crucial effect on the characteristics of the materials [73,74,75]. Currently, the scientific literature is rich in publications reporting the morphological tuning of NPs, ranging from metallic ones to organic ones [76,77]. In this context, PDA was not excluded; the versatility of this material and the synthetic strategies allow for a great control over its morphology [78]. In fact, several protocols have reported the possibility of producing spherical and mesoporous PDA nanoparticles (MPDA NPs), PDA nanocapsules, and also PDA homogenous layers, as the coating of surfaces or films [79,80]. Herein, we will focus on the way in which the shape of PDA could affect the performances of this biomimetic material in drug delivery applications, and we will go into detail about drug loading, drug release, and cellular uptake, trying to highlight the pros and cons of each morphology. Regarding the synthetic procedure that concerns the polymerization of PDA, spherical nanoparticles (PDA NPs), MPDA NPs, PDA nanocapsules, and molecular coatings (MCs) can be obtained following different protocols. The synthesis of PDA NPs is mainly performed by stirring an alkaline aqueous solution of dopamine; Ju et al. reported the formation of PDA NPs by mixing dopamine in a NaOH/water solution [81], while Xiao et al. reported the assembly of PDA NPs combining dopamine with a NH_3_/ethanol/water mixture [82]. The former method is characterized by a major product yield, while the latter guarantees a better colloidal stability of the nanoparticles and a more monodispersed size. Concerning MPDA, Tang et al. presented a facile strategy to obtain highly porous nitrogen-rich carbon nano-spheres. Briefly, they designed a self-polymerization process of dopamine and a spontaneous co-assembly of diblock copolymer micelles [83]. The synthesis of PDA nanocapsules, first developed by Caruso et al. in 2011 [84], is based on the coating of a PDA layer on the surface of a template, such as silica, MOFs, polystyrene, or emulsion droplets, followed by the template removal [85,86]. The kind of template and the reaction conditions allow for tuning the size of the capsules and the thickness of the PDA shell [86]. As we mentioned before, PDA has been exploited not only in the form of NPs, but also as PDA-based coatings and films that have become very popular strategies to provide biocompatibility, cellular uptake, and antioxidant properties to biomaterials employed in the biomedical field [50,87]. Usually, the functionalization of surfaces is performed by simply dip-coating the heterogenous substrates in an alkaline aqueous solution of dopamine, forming a homogeneous layer of PDA [88], while films are usually produced by the casting of blends based on polymeric materials and PDA [89] (Figure 5).

Talking about drug delivery, MPDA NPs have been widely explored as promising platforms for the transport of drugs in body fluids. The most appreciated characteristic of this class of particles is the very high surface-to-volume ratio, which usually allows for major quantitative uptake of pharmaceutical active ingredients (PAIs) inside the NPs compared to non-porous delivery systems [91]. Despite the fact that this is well-reported for nanomaterials like silica [92] and MOFs [93], the effects of porosity on the efficiency of drug delivery platforms based on melanin-like materials is just at the very beginning. On that topic, Chen et al. have compared the efficiency of PDA NPs and MPDA NPs as potential drug carriers [94]. They reported the formulation of an antioxidant and photoprotective nanoplatform for the dispatchment of a photosensitive PAI, known as retinoic acid (RA). In analogy with the aforementioned materials, MPDA NPs had absorbed a major quantity of RA compared to PDA NPs, reaching a concentration of RA of about 90 μg∙mL^−1^ in the product. Long incubation time and elevated drug:carrier quantitative ratio had also contributed to enhancing the drug loading. In addition, cells treated with RA-loaded MPDA NPs demonstrated higher cellular uptake than RA-loaded PDA NPs. The superior loading capacity of MPDA NPs was also exploited to internalize more than one chemical per instance to design theranostic platforms. For example, Shu et al. have presented a multifunctional platform for cancer treatment [95]. MPDA NPs were loaded with doxorubicin (DOX), a potent chemotherapeutic drug, and iron oxide (IO), a magnetic resonance active species (MRAS); in addition, NPs were decorated with sialic acid to provide a specific hepatic targeting. MPDA NPs not only guaranteed a great drug loading (up to 40%), but also photothermal and photo-release properties, and pH-triggered liberation of DOX into the acid of the malignant cell’s cytoplasm. However, drug loading efficiency decreased by increasing drug feeding; this highlights that the saturation level of MPDA NPs, as a drug carrier, is a crucial parameter to be investigated to reach the desired drug loading by employing a minor amount of drug. Another interesting fact is that high loading capability also facilitates the design of cures where the synergetic work of different chemicals is needed. On that topic, interesting IO-loaded MPDA NPs were also described by Guan et al. [96]. In this case, IO was chosen as a metal supplier to induce ferroptosis in tumor cells. Furthermore, the pores of MPDA NPs were also loaded with sorafenib (SRF), a ferroptosis inducer, and glutathione, which favors the release of SRF, to increase to efficiency of the treatment. Indeed, the co-loading of two or more substances on the same carrier is a promising approach to exploit the cascade effect of multiple reactions developing efficient medical treatment. For example, Ren et al. aimed to utilize gas therapy for cancer treatment; thus, they functionalized MPDA NPs with arginine and IR780, a photosensitizer for ROS production [97]. Indeed, radical species can degrade the amino acid into gaseous nitric oxide (NO). NIR irradiation not only induced the formation of ROS and NO, but also permitted researchers to perform photoacoustic imaging and also promoted drug release and the cellular uptake of arginine and IR780 due to the photothermic properties of PDA. Here, it is interesting to notice that pH had the opposite effect on the release of arginine and IR780, whereas NIR irradiation drastically promoted the administration of both chemicals. This phenomenon underlines how photo-release should be taken into consideration as a primary approach in stimuli-responsive PDA nanoparticles. PDA nanocapsules are another example of PDA NPs that enhance the drug delivery performances thanks to their morphology. As in the case of MPDA NPs, PDA nanocapsules have higher surface-to-volume ratio with respect to bare NPs, enhancing drug loading. A peculiar property of PDA hollow NPs is also the possibility of loading the drug during their synthesis. A first example was reported in the work of Cui et al. [98]. They obtained PDA nanocapsules using dimethyldiethoxysilane emulsion droplets as the template; they managed to load the hydrophobic drug thiocoraline directly in the emulsion droplets before PDA synthesis, and the drug remained encapsulated even after the template removal. Ding et al. synthesized PDA nanocapsules using an arginine-modified linoleic acid as the template emulsion [99]. They performed the co-loading of two drugs in this system. The hydrophobic drug Paclitaxel was encapsulated during PDA NP synthesis, while DOX (hydrophilic) was anchored at the PDA surface by π-π stacking interactions. The synergy between the two drugs led to a higher performance in killing Hep-G2 cells, with respect to the PDA nanocapsules loaded with one of the two drugs. Another interesting example is reported by Wong et al., who obtained PDA nanocapsules templated by the drug itself (curcumin). Indeed, they managed to stabilize curcumin colloidal aqueous solution with fructose. This represented the template for the PDA coating layer formation. After a dialysis process, fructose was removed and PDA nanocapsules with a very high loading of curcumin (93–97%) were obtained. Moreover, it was also possible to efficiently embed other hydrophobic drugs with curcumin, such as Albendazole and Sulfasalazine. As we have discussed so far, nanoparticles themselves could guarantee a targeted treatment of localized diseases like solid tumors. However, free nanoparticles do not fit as well as platforms for drug delivery when large areas must be treated, like in dermal wounds or implant coating. In these cases, molecular coating and patches are preferred because their morphology is better suited when a wider and more homogeneous coverage is required. In addition, recently, PDA coatings have been successfully applied as osteogenic materials in orthopedic implants [100]. As for nanoparticles, the tailoring of the fabrication strategies of the biomaterials can lead to a broad variety of outcomes. For example, Türk et al. proposed a gentamicin-loaded PDA coating as a promising strategy to prevent titanium implant-related bacterial infections after surgery [101]. They focused on the strategies to load the drug onto the coating and their effect as an antibacterial material. The study highlighted that mixing gentamicin with PDA before the application on the implant demonstrated superior properties. Indeed, the attempts to absorb the drug on already-formed PDA coating demonstrated modest effects against microbial infections. Recently, studies went beyond the coating strategy; in fact, the antibacterial properties of PDA and its efficient drug delivery were also exploited to design drug delivery platforms in a film shape for lesion treatment. On that topic, Tao et al. loaded MPDA NPs onto a chitosan/hyaluronic acid composite material to create a photo-responsive patch for the disinfection of injuries [102]. Curcumin was uptaken into MPDA NPs before their incorporation into the polymeric film. The authors claimed that NIR irradiation was crucial to obtain the best performance; indeed, antibacterial assays confirmed that irradiated samples presented significant antibacterial abilities against both Gram-negative (*E. coli*) and Gram-positive (*S. aureus*) bacteria via photothermal effect and accelerated the release of curcumin. In summary, we can say that PDA is a very versatile material that can be shaped in a very broad range of different morphologies, and because of that, we should select the best form depending on our aim to reach the most promising results.

## 4. Influence of Surface Charge

**Figure 6:**
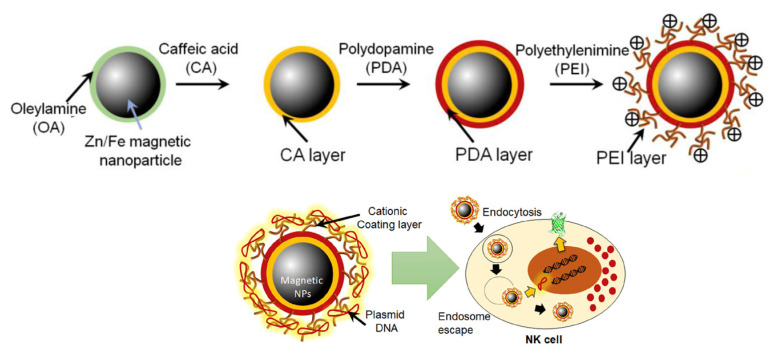
Synthesis of the multi-functional NPs and their delivery mechanism. Adapted with permission from ref [115], copyright 2019, Elsevier.

Surface charge is a particularly important property of PDA NP use in drug delivery since it strongly influences three aspects: drug release mechanism, cellular uptake, and blood circulation. The presence of catecholic and amino groups gives PDA amphoteric properties; at high pH, they present a negative surface charge because of catechol deprotonation, and a low pH, they are positively charged, thanks to amino group protonation [103]. This pH-dependent behavior is the key factor on which the pH-induced delivery is based. Indeed, the drug can remain stable and linked to the NPs during blood circulation and be released in the acidic TME [104]. For example, this happens in the case of DOX loading; at pH 7.4, DOX can be efficiently loaded into PDA NPs, while in acidic conditions, the protonation of the amino groups increases the DOX solubility, triggering its delivery [105]. As far as concerns cellular uptake, surface charge is one of the driving forces that govern the interaction between cells and nanosystems. Indeed, the cell internalization pathway starts with the binding of the nanoparticle with the cell membrane, affected by NP surface charge [106,107,108]. Usually, sulfate proteoglycans are present on the surface of the cell, conferring negative charge [109,110]; thus, the electrostatic interaction between the anionic cell surface and cationic nanoparticles favors binding and subsequent internalization [111]. Moreover, negatively charged NPs are often opsonized and cleared by macrophages in the reticuloendothelial system [112]. For these reasons, PDA NPs, which have a negatively charged surface, are often coated with polymers to decrease the absolute value of their negative zeta potential. For example, Lin and co-workers developed a theranostic system in which Prussian blue (PB) NPs were coated with PDA for the efficient loading and pH-triggered release of DOX and were then further functionalized by PEG and folic acid (FA) to increase, respectively, physiological stability and tumor targeting [113]. The zeta potential of the PDA-coated NPs was −18.4 mV, with PEG increased to −12.5 mV. The addition of FA had the role of binding with the folate receptors expressed on the tumor cells. Its presence decreased the zeta potential to −17.5 mV because of the negative charges of folate groups. However, this allowed an efficient cellular uptake; this was due to FA targeting ability, but also to the stabilization given by the PEG shell, without whom the charge would have probably been even more negative. Another example of PDA modification by PEG is given by Zheng and co-workers [114]. They developed a Ca^2+^ nanogenerator for use in Ca^2+^-overload-mediated cancer therapy, embedded with cisplatin (CDDP) and curcumin (CUR). In this work, CaCO_3_ NPs were coated with PDA and, in order to favor the circulation and the cell uptake, a further coating of an acidity-sensitive mPEG-DMDF (2,5-Dihydro-2,5-dimethoxyfuran) was performed. This modification allowed for an increase in the zeta potential from −15 mV to −3 mV. The NP decomposition was tested at pH 7.4, 6.8, and 5.5; the NPs maintained their stability at pH 7.4, partially decomposed at pH 6.8, and had a total decomposition at pH 5.5, triggering drug release of 88.0%, 71.3%, and 63.8% for Ca^2+^, CUR, and CDDP, respectively, after 6 h. Cellular uptake displayed an effective internalization for 4 h post-injection and an efficient tumor accumulation was also observed in MCF-7-tumor-bearing mice. Results confirmed that the PEG modification of these nanosystems contributed to enhancing water solubility, blood circulation, and tumor accumulation. Kim et al. developed a PDA-based nanosystem for the genetical manipulation of natural killer (NK) cells, lymphocytes involved in the immune surveillance of malignant and pathogen-infected cells [115]. NPs with a Zn/Fe magnetic core were synthesized and then coated with a PDA layer. The use of PDA allowed them to functionalize the system with polyethyleneimine (PEI) to have a cationic surface (Figure 6).

**Figure 7:**
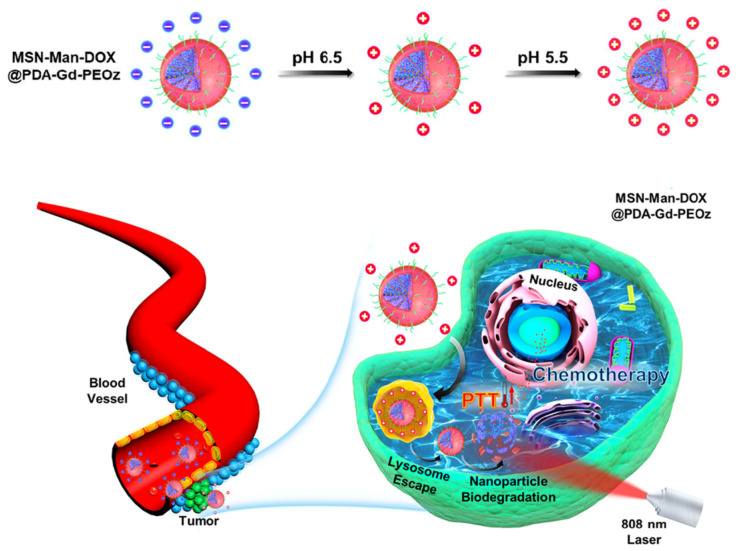
Surface charge pH-dependent variation in mannose-doped MSN loaded with doxorubicin and coated with PDA and gadolinium, functionalized by PEOz (MSN-Man-DOX@PDA-Gd-PEOz), and schematization of the treatment process. Adapted with permission from ref [116], copyright 2021, Elsevier.

Indeed, after the modification with PEI, PDA-coated NPs had an increase in the zeta potential from −21 mV to +35 mV and exhibited good stability both in PBS and cell culture medium. Moreover, PEI functionalization allowed NPs to have a cationic surface for an efficient binding of a DNA plasmid for their delivery in the immune cells (NK), as shown in Figure 6. NPs’ activity as gene carriers was first evaluated in vitro, in NK-92MI cells, observing their distribution by a bio-TEM. NPs were found both in cellular membrane and endosomes, confirming their successful uptake. Also, the delivery of the genetic material into the NK cells was effective. In the work of Li et al., PDA was functionalized with poly (2-Ethyl-2-Oxazoline) (PEOz) in a system for tumor therapy, in order to facilitate tumor penetration [116]. They developed mannose-doped mesoporous silica NPs (MSN) loaded with doxorubicin and coated with PDA and gadolinium (Gd^3+^), obtaining a drug delivery system with photothermal properties for chemo/photothermal therapy and a contrast agent for MRI, thanks to the presence of Gd^3+^. The nanosystem was then modified by PEOz, a long-chain hydrophilic polymer, with tertiary amines in its structure [117]. As already mentioned, during blood circulation, a negative surface charge can be beneficial, since it avoids the interaction with negative charge proteins and other factors which would induce the removal by the endothelial network system [118], while a positively charged surface can help cell internalization. The presence of PEOz led to a negative charge on the surface at pH 7.4 (Zeta potential −33.7 mV), while this charge was completely reversed at acidic pH, thanks to amine protonation (zeta potential +15.7 mV at pH 6.5 and +33.7 mV at pH 5.5) (Figure 7).

Moreover, even if PDA usually has a negative surface charge, in this case, gadolinium doping led to a positive zeta potential (+27.5), so the presence of PEOz was required to restore a negative charge at neutral pH. Cellular uptake was tested with A549 tumor cells, resulting in an effective uptake after 3 h of incubation. The performance of PEOz-functionalized NPs was also compared to that of PEG-modified NPs, in which no positive charge was observed at acidic pH, and highlighted the superior properties of PEOz functionalization. Instead, Xiong and co-workers used a hydroxyethyl starch (HES)-based prodrug to obtain PDA nanosystem stabilization [119]. They synthesized Cu^2+^-doped PDA NPs (copper has the role of enhancing PDA photothermal properties) and they modified their surface with HSD, a HES-based redox-sensitive prodrug of DOX. Indeed, they reported in a previous work that HES could improve the stability of PDA in a physiological environment [120]. Indeed, the HSD-modified NPs showed great stability compared to the unmodified ones, remaining unchanged even after one month of storage. Moreover, the zeta potential increased from −19.3 mV to −10.7 mV after HSD modification, favoring cellular uptake and maintaining a good blood circulation. The pharmacokinetics of the system was evaluated in mice; stability given by HSD favored both the blood circulation and guaranteed an effective internalization in tumor cells. Alternatively, Zhang et al. enhanced the pharmacokinetics of PDA-based NPs by camouflaging them with a stem cell membrane [121]. They synthesized PDA NPs and loaded them with SN38, a hydrophobic drug that interacts with PDA by means of π-π stacking interactions [122]. They coated the SN38-PDA NPs with stem cell membranes (SCM), which allowed the system to have a longer blood circulation (avoiding macrophage uptake) and accumulate in the tumor site more effectively. The zeta potential of SCM-modified PDA NPs (PDA@SCM NPs) increased to −28.4 mV (from −43.5 mV); this value was comparable to those of SCM vesicles (−32.5 mV) with a confirmed SCM coating and, while maintaining the negative charge, helped the blood circulation. To better investigate this aspect, the phagocytic activity of macrophages on PDA@SCM NPs was evaluated by flow cytometry, using RAW246.7 macrophages. After 12 h of incubation, 65.9% of PDA@SCM NPs were internalized by the macrophages, a lower uptake than that of PDA NPs (93.0%), confirming the camouflaging action of SCM. PDA-SN38@SCM NPs were internalized in 91.9% of the cancer cells, while PDA-SN38 NPs (without SCM) were present in 73.5% of the cells; this confirmed the targeting ability of SCM in this system. Finally, blood retention and distribution of PDA-SN38@SCM NPs was assessed in major organs and tumor tissues, confirming higher blood retention and tumor accumulation in comparison with PDA NPs not camouflaged by SCM. These examples showed that there are many possibilities to functionalize PDA NPs, to improve their blood distribution and cellular uptake. In general, as already mentioned, negative surface charge, which is one of the main properties of PDA NPs, can be beneficial for the blood circulation, because it avoids interaction with proteins and other agents present in blood. On the other hand, it has been reported that a negative surface charge can lead to opsonization or internalization by macrophages in the reticuloendothelial system. Moreover, a negative charge on the surface is not favorable for an efficient cell internalization. In line with these considerations, the PDA surface is usually modified to obtain a less negative zeta potential, while still maintaining a negative charge. This is a good compromise between efficient blood circulation and cellular uptake, which always improves PDA NPs’ pharmacokinetics. The other PDA charge-related property which is important for drug delivery is the amine protonation that occurs at acidic pH and leads to drug release in TME. Even with functionalization of the PDA surface, this effect must be (and usually is) maintained.

## 5. Future Perspectives

### 5.1. Size and Morphology Dependence Investigation

As described in Section 2 and Section 3, size and morphology are two of the fundamental parameters that govern drug delivery-related mechanisms. Indeed, they influence the cellular uptake pathway, drug loading, and drug release. In the view of a controlled PDA design for delivery purposes, it would be beneficial to have a systematic study of PDA size and morphology influence. In a drug delivery system, based on the agent to be released and the kind of therapy to be performed, it could be useful to control the pharmacokinetics and the releasing time. This kind of investigation on PDA size and morphology would help in designing the proper PDA system, based on the conditions that must be achieved.

### 5.2. Exploration of Other Melanin-Based Materials for Surface Effects

Section 4 describes how surface charge influences drug delivery processes in PDA NPs. Charge is important in the blood circulation of the system and in cell internalization. Moreover, it also has a role in the pH-triggered release of the drug. As far as concerns the pharmacokinetic aspects, PDA surface charge is often not favorable. Indeed, PDA has a quite negative surface charge, and it does not help either blood circulation (for which a less negatively charged surface would be more advantageous) or internalization in the cells (which would be favored by a positive surface charge). Consequently, PDA is often modified by means of functionalization strategies, in order to change its surface properties. PDA nanosystems used for delivery purposes are the ones typically synthesized by oxidative self-polymerization, but it would be interesting to use also other kinds of melanin. Indeed, natural melanin is synthesized in many organisms and is present in different forms: eumelanin, pheomelanin, allomelanin, neuromelanin, and pyomelanin [123]. These melanin classes depend on the nature of the polymerization precursors and, even if the structure is almost the same, they usually present some differences, such as surface properties, because the functionalities present on the surface depend on the precursors at the basis of the synthesis. Thus, it would be beneficial to compare the surface charge properties of the distinct kinds of melanin and investigate their roles for drug delivery purposes.

## 6. Conclusions

PDA is a very versatile material widely used in biomedical applications. Currently, there is a lot of research concerning PDA-based nanomaterials for drug delivery. Indeed, PDA has peculiar properties, such as biocompatibility and ease of functionalization, that make it a proper platform for this purpose. Moreover, PDA is able to release agents upon exposure to two stimuli: NIR irradiation, exploiting its photothermal properties, and acidic pH stimulus, exploiting amine group protonation. In this review, PDA behavior in drug delivery was analyzed based on PDA size, morphology, and surface charge. Indeed, these three characteristics have a huge influence on PDA-mediated drug loading and release and on NP blood circulation and uptake in the cells. The deeper study of size, morphology, and surface charge-related phenomena can help to design new PDA-based nanomaterials for specific applications in advanced drug delivery systems.

## Data Availability

No new data were created or analyzed in this study. Data sharing is not applicable to this article.
